# EuroSCORE II: Current limitations and physiological gaps in risk stratification

**DOI:** 10.1113/EP092900

**Published:** 2025-07-22

**Authors:** Jing Yong Ng, Eu Fon Tan, Marsioleda Kemberi, Eduardo Urgesi, Matti Jubouri, Damian M. Bailey, Mohamad Bashir, Wael I. Awad

**Affiliations:** ^1^ Barts Heart Centre St Bartholomew's Hospital London UK; ^2^ Barts Health NHS Trust Milton Keynes University Hospital, Eaglestone Milton Keynes UK; ^3^ Hull York Medical School University of York York UK; ^4^ Neurovascular Research Laboratory, Faculty of Life Sciences and Education University of South Wales Cardiff UK; ^5^ William Harvey Research Institute Queen Mary University of London London UK

**Keywords:** cardiac surgery, EuroScore, risk evaluation, risk modelling, risk stratification

## Abstract

Risk stratification remains critical in cardiac surgery, enabling clinicians to predict adverse outcomes and guide perioperative management. The European System for Cardiac Operative Risk Evaluation (EuroSCORE) II, introduced in 2011, incorporates 18 key variables to provide an evidence‐based approach to risk assessment. However, evolving surgical techniques, changing patient demographics, and emerging evidence reveal limitations in the model's predictive capabilities. Important factors such as frailty, race, liver dysfunction, left ventricular dimensions, and advanced cardiac function metrics are not incorporated, reducing its accuracy in diverse and high‐risk populations. Additionally, the model does not fully account for key conditions, such as infective endocarditis, where high‐risk features like embolic events and abscesses significantly impact surgical outcomes. Simplified categorisation of procedures and the binary assessment of coronary artery disease overlook critical complexities, such as lesion severity and procedural variability. Advanced parameters like global longitudinal strain (GLS), SYNTAX, and Model for End‐Stage Liver Disease (MELD) scores could enhance the model's granularity and predictive power. Furthermore, integrating machine learning into future iterations of EuroSCORE offers the potential to capture non‐linear interactions and continuously refine predictions. These updates could pave the way for a ‘EuroSCORE III’ better aligned with modern surgical practices, offering improved precision in risk stratification, more personalised clinical decision‐making and optimised patient outcomes.

## Abstract (highlights)

- **What is the topic of this review**?
  The evolution of surgical practices and patient demographics over the last decade highlights the need for further refinement of the EuroSCORE model to address its limitations and reflect modern practices. Key patient‐related factors as well as procedural complexities, are absent in EuroSCORE II.
- **What advances does it highlight**?
  EuroSCORE II oversimplifies operation‐related factors by grouping procedures or combined surgeries under single categories. This lack of granularity impacts its utility for personalised surgical planning and outcome prediction. Leveraging ML can enhance the predictive accuracy of future iterations of the EuroSCORE model due to its ability to capture non‐linear relationships and integrate complex variables.

## 1 INTRODUCTION

Risk stratification is a statistical tool used to identify patient characteristics associated with an increased likelihood of adverse clinical outcomes (Jackson, 2001). By pinpointing these risk factors, clinicians can implement targeted interventions to mitigate their impact and optimise patient care (Jackson, 2001). Cardiac surgery was amongst the first specialities to adopt risk stratification tools, given the high‐risk nature of its procedures and the need for standardised assessments to improve patient outcomes. Early models, such as the Parsonnet score introduced in the 1980s, pioneered the estimation of surgical mortality by assigning weighted scores to patient‐specific factors, including age, comorbidities and the urgency of surgery (Granton & Cheng, 2008). Building on this foundation, subsequent models, like the Society of Thoracic Surgeons (STS) score and the European System for Cardiac Operative Risk Evaluation (EuroSCORE), were developed to address the limitations of earlier models and incorporate advancements in data collection and statistical modelling (Granton & Cheng, 2008).

The original EuroSCORE, developed in 1999, featured additive and logistic versions, each employing different calculation methods for distinct applications. The additive EuroSCORE assigned fixed points to various risk factors, summing them to produce an overall risk score (Nashef et al., 1999). While straightforward, this version was criticised for its simplistic approach, as it did not account for interactions between risk factors, particularly in high‐risk patients, often leading to an underestimation of mortality in complex cases. The logistic EuroSCORE was developed to address these limitations, utilising a more sophisticated algorithm capable of modelling non‐linear relationships between variables and improving predictive accuracy for high‐risk patients (Roques et al., 2003). However, advances in medical practices and shifts in patient demographics eventually exposed limitations in the logistic model's reliability as a model that overpredicts risk.

EuroSCORE II, the most recent iteration of the EuroSCORE model, was introduced in 2011 following a comprehensive evaluation of data from 22,381 patients undergoing major cardiac surgeries across 154 hospitals in 43 countries (Nashef et al., 2012). This updated model incorporated a broader range of variables compared to EuroSCORE I while retaining 14 key factors, some of which were modified. Examples include creatinine clearance, left ventricular (LV) function, systolic pulmonary artery pressure, urgency and weight of operation – selected for their statistically significant association with mortality, identified primarily through logistic regression and refined using advanced statistical techniques. EuroSCORE II provides a more accurate model‐predicted mortality rate of 3.9% compared to 4.6% while maintaining strong discrimination (Nashef et al., 2012). Although it demonstrated significant improvements in risk stratification, aligning with advancements in perioperative care, surgical techniques and patient management at its introduction, over a decade has passed. The field of cardiac surgery has continued to evolve, prompting several studies to reassess the model's reliability in the context of changing variables and its role as a predictor of perioperative mortality risk (Hirnle et al., 2024; Mastroiacovo et al., 2022).

This paper critically analyses the three main domains of EuroSCORE II – patient‐related factors, cardiac‐related factors and operation‐related factors – highlighting the limitations of the current variables and the impact of excluded factors on its predictive performance (Table 1). It aims to inform the development of a future ‘EuroSCORE III’ model, designed to serve as a more effective tool for risk stratification, support enhanced clinical decision‐making, improve outcome prediction and optimise patient care.

**TABLE 1:** Summary of current EuroSCORE II limitations across key domains.

Domain	Limitations
Patient‐related factors	Absence of frailty as a predictor of surgical risk
	Lack of inclusion of ethnicity
	Binary categorisation of endocarditis, overlooking the spectrum of the disease
Cardiac‐related factors	Exclusion of liver dysfunction metrics
	Overreliance on LVEF, which can misrepresent myocardial infarction in certain patient populations
	Lack of consideration of LV dimensions as predictor of outcomes
	Binary treatment of CAD, failing to address lesion complexity, vessel involvement or anatomical challenges
Operation‐related factors	Oversimplification of procedural complexity (e.g., mitral valve replacement and repair carrying the same weight)
	Does not differentiate risks for combined procedures
	Omission of high‐risk operations such as pericardiectomy

Abbreviations: CAD, coronary artery disease; LV, left ventricle; LVEF, left ventricular ejection fraction.

## 2 PATIENT‐RELATED FACTORS

### 2.1 Frailty

Frailty is a multifactorial clinical syndrome characterised by an increased vulnerability to minor stressors as a result of diminished physiological reserve (Rowe et al., 2014). In cardiac surgery, frailty strongly predicts poor outcomes. A systematic review and meta‐analysis found that frail patients undergoing cardiac surgery had twice the adjusted operative mortality of non‐frail patients (Lee et al., 2021). Frailty is also linked to higher rates of postoperative complications, including delirium, cardiorespiratory events (such as myocardial infarction and heart failure), wound infections and bleeding (Lee et al., 2010, 2021; Pozzi et al., 2023). These complications contribute to prolonged hospital stays, increased dependency on rehabilitation or non‐home facilities, and diminished long‐term survival (Lee et al., 2021).

The adverse effects of frailty stem from its underlying pathophysiology, which includes chronic inflammation, neurohormonal dysregulation, sarcopenia and immune dysfunction – all of which impair the body's ability to tolerate surgical stress and recover effectively (Ijaz et al., 2024; Soysal et al., 2016). Despite its significant impact, frailty remains an underutilised factor in cardiac surgical risk assessment. Incorporating frailty into risk stratification could improve patient selection, helping to identify individuals who would benefit from intervention while avoiding unnecessary procedures in those unlikely to tolerate aggressive treatment.

A consensus statement from six major international, European and United States societies recommends frailty screening for all patients aged 70 years and older, yet no single tool has been universally adopted, reflecting its complex and multifaceted nature (Afilalo et al., 2017; Morley et al., 2013).

While reduced mobility has long been a binary variable in EuroSCORE, introduced in EuroSCORE I and defined as ‘an effect whether due to neurological dysfunction or musculoskeletal dysfunction’, it serves only as a surrogate marker for frailty rather than a comprehensive assessment (Nashef et al., 2012; Redmond et al., 1996). The subjective nature of mobility assessment introduces variability between clinicians, reducing its reliability as a frailty measure. Moreover, reduced mobility alone does not fully encapsulate the complexity of frailty, highlighting the need for a more objective measure to replace or supplement this variable in risk models.

The FRAILTY‐AVR study by Afilalo et al. identified frailty as a crucial predictor of adverse outcomes following aortic valve replacement (TAVR/SAVR) (Afilalo et al., 2017). The study found that the Essential Frailty Toolset (EFT) outperformed other frailty measures, including the Fried Frailty Phenotype and Rockwood Clinical Frailty Scale (CFS), in predicting mortality and disability. Unlike reduced mobility, the EFT provides a multidimensional assessment, incorporating functional, cognitive and biological parameters such as lower‐extremity weakness, cognitive impairment, anaemia and hypoalbuminemia, leading to more accurate risk stratification (Afilalo et al., 2017).

### 2.2 Race

Race influences surgical outcomes, with disparities in disease prevalence, comorbidities and postoperative complications. Black patients undergoing coronary artery bypass grafting (CABG) have 1.5 times higher early mortality risk than non‐Black patients, with the gap worsening over time (Khera et al., 2015). A meta‐analysis found that Black patients undergoing CABG have higher rates of preoperative comorbidities, in‐hospital mortality and postoperative complications, including pneumonia, stroke, bleeding, renal failure requiring dialysis and respiratory failure necessitating tracheostomy, compared to White patients (Benedetto et al., 2019).

Additionally, Asian populations exhibit distinct cardiovascular risk profiles, including a higher prevalence of diabetes mellitus, more diffuse coronary artery disease (CAD) and smaller coronary vessels (kanaya, 2024; Kooner et al., 2024; Skowronski et al., 2020). These factors contribute to accelerated atherosclerotic disease progression, greater technical challenges during grafting and reduced graft patency in CABG, ultimately influencing long‐term surgical outcomes (Park et al., 2022; Zhang et al., 2021). While 1‐year survival rates for isolated cardiac surgery in South Asian patients are comparable to White patients, they face a 24% higher mortality risk for combined CABG and valve surgery and a 27% greater likelihood of heart failure‐related admissions (Lai et al., 2024).

A study comparing the STS and EuroSCORE II models found that STS provides better discrimination and calibration, particularly for Asian populations. It also showed that EuroSCORE II reclassified 31% of STS low‐risk patients into intermediate or high‐risk categories, raising concerns about potential risk overestimation in diverse populations (Seto et al., 2022).

Incorporating ethnic and racial factors into the EuroSCORE II, as exemplified by the STS risk score, could enhance its ability to account for the unique risks faced by diverse populations across Europe. Such an improvement would strengthen the model's accuracy, promote equitable risk assessment and improve patient outcomes while addressing disparities in surgical care.

### 2.3 Infective endocarditis

The incidence of infective endocarditis (IE) is rising, with approximately half of affected patients requiring cardiac surgical intervention (Thornhill et al., 2020). Surgery is often essential for managing complications that cannot be adequately addressed through medical therapy alone. Despite advancements in diagnosis and treatment, IE remains a life‐threatening condition with a 1‐year mortality exceeding 30% (Talha et al., 2021). Active IE is a well‐established risk factor for mortality in major cardiac surgery, making it an integral component of EuroSCORE II.

However, the predictive accuracy of EuroSCORE II for patients with IE undergoing cardiac surgery remains controversial. EuroSCORE II classifies active IE as a binary variable, oversimplifying a condition with a broad spectrum of disease severity and associated complications. A study by Varela et al., 2018, found that EuroSCORE II underestimates surgical mortality in IE patients, likely due to the absence of IE‐specific risk factors such as abscess formation and septic complications. In contrast, a recent nationwide validation study by Heinen et al. reported that EuroSCORE II overestimates mortality when the predicted risk exceeds 20%, indicating that for high‐risk IE patients, actual mortality may be nearly half the predicted value (Heinen et al., 2024). Despite these discrepancies, both studies agree that EuroSCORE II lacks optimal calibration for IE, highlighting the need for risk models that incorporate IE‐specific variables. For example, STS‐IE and PALSUSE scores have shown superior predictive accuracy, offering better discrimination and calibration for this high‐risk population (Varela et al., 2018).

Amongst the many complications of IE, embolic events occur in 40–50% of cases, presenting as ischaemic or haemorrhagic strokes, peripheral emboli (splenic, renal), pulmonary embolism and coronary artery embolism (Bhattacharyya & Oo, 2019). Li et al. (2022) reported that IE patients with systemic embolism undergoing open‐heart surgery for left‐sided IE face a significantly higher risk of mortality compared to those without embolic events. Similarly, García‐Cabrera et al. (2013) found that IE patients who underwent valve surgery and had neurological complications had an early postoperative mortality rate of 45%, compared to 24% in those without. These complications included moderate‐to‐severe ischaemic events and cerebral haemorrhage, including primary intracerebral haemorrhage and haemorrhagic transformation of ischaemic strokes. Notably, valve surgery outcomes were better for patients with embolic strokes than for those with haemorrhagic strokes.

Another severe IE‐related complication is cardiac abscess formation, with perivalvular abscess occurring in 30–40% of cases, most frequently involving the aortic valve (Ramos Tuarez et al., 2024). These abscesses are associated with a higher risk of conduction abnormalities, embolisation and aortocavitary fistula formation, making early surgical intervention critical to improving patient outcomes. However, recent evidence suggests that abscess presence significantly increases short‐term surgical mortality, likely due to greater disease severity necessitating more complex procedures (Degife et al., 2024; Ramos Tuarez et al., 2024; Yang et al., 2021). Furthermore, it has also been shown that aortic valve replacement and aortic root replacement for IE complicated by periannular abscess are associated with a high risk of cardiac‐related complications. These include elevated mortality rates (especially within the first year), paravalvular leaks and the need for valve reoperations (Ting et al., 2020).

The binary classification of IE in EuroSCORE II fails to capture the condition's heterogeneity, overlooking key factors such as systemic involvement, structural complications and infection status. This limitation leads to inaccurate risk predictions, particularly in patients with complex pathology like systemic embolism and abscess formation.

Patient‐related factors such as frailty, race and IE play a crucial role in shaping cardiac surgical outcomes. However, EuroSCORE II's failure to adequately account for these variables highlights the need for more refined risk models that incorporate frailty screening, racial disparities and IE‐specific complications to enhance risk prediction, surgical decision‐making and patient outcomes. Given the diverse demographic and clinical profiles of the contemporary patient population, there is an increasing need to broaden the scope of patient‐specific factors in EuroSCORE II to improve the precision and personalisation of risk assessments.

## 3 CARDIAC‐RELATED FACTORS

### 3.1 Liver dysfunction

Liver dysfunction significantly affects outcomes in cardiac surgery patients, yet it remains unaccounted for in widely used risk models, including the EuroSCORE II (Hawkins et al., 2019; Nashef et al., 2012). In particular, patients with liver cirrhosis undergoing cardiac surgery represent a high‐risk group due to the complex interplay of liver dysfunction with cardiac surgery outcomes, which underscores the importance of accurately assessing this risk in predictive models.

While liver dysfunction was considered during the development of EuroSCORE, it was ultimately excluded after serum albumin – a widely available marker of hepatic dysfunction – failed to demonstrate a significant relationship with surgical risk (Nashef et al., 2012). As noted in the EuroSCORE paper, the measurement of serum albumin presented challenges, including variability in units and assay techniques across centres, which may have undermined its predictive utility. Unfortunately, despite the recognition that liver dysfunction increases cardiac surgery mortality, no alternative indicators were explored, leaving this critical risk factor unaccounted for in the model.

Recent studies highlight the considerable impact of liver dysfunction on perioperative and long‐term outcomes in cardiac surgery. Lin and Hsu (2014), for example, demonstrated that patients with advanced liver cirrhosis face significantly higher mortality, with preoperative serum bilirubin levels being a strong predictor of both early and late mortality. Additionally, in 2022, a meta‐analysis involving 48,891 patients undergoing open‐heart surgery highlighted the association between liver dysfunction and poorer outcomes. In this study, 18,979 patients with high liver dysfunction scores, based on Child–Pugh or Model for End‐Stage Liver Disease (MELD) scores, exhibited markedly increased rates of perioperative mortality, neurovascular complications, prolonged ventilation, sepsis, acute kidney injury and long‐term mortality (Jacob et al., 2015; Kirov et al., 2022). These findings highlight the pervasive influence of liver dysfunction on surgical outcomes and the critical need for its integration into risk prediction models.

Impaired coagulation is another major challenge for cirrhotic patients, as liver dysfunction compromises the synthesis of clotting factors. Cardiac surgery procedures, especially those involving cardiopulmonary bypass, exacerbate these haematological issues through mechanisms like hypothermia, haemodilution and hypoperfusion. Such complications make cirrhotic patients more vulnerable to adverse outcomes, necessitating careful perioperative management.

The MELD score is a numerical scale used to assess the severity of chronic liver disease based on laboratory values such as bilirubin, International Normalized Ratio (INR), the time it takes for blood to clot, and creatinine and is commonly employed to predict short‐term mortality risk. A MELD score of 9 or higher increases cardiac surgery mortality risk 2‐ to 5‐fold, illustrating the substantial influence of even moderate liver dysfunction on postoperative survival (Lopez‐Delgado et al., 2022). A recent study investigating liver dysfunction and cardiac surgery outcomes further stresses this impact, showing that 18.8% of cardiac surgery patients have a MELD score above 10, indicating a significant prevalence of underlying liver dysfunction (Pathare et al., 2023). Failure to incorporate this factor into EuroSCORE II could result in underestimating risk for a significant subset of patients, thereby reducing the model's predictive accuracy.

Although not all cirrhotic patients face uniformly high risks, the mortality risk remains considerable, particularly amongst those with more advanced liver disease. While patients in Child–Pugh class A may have a relatively lower risk of mortality within the first 30 days post‐surgery compared to those in classes B or C, the long‐term survival outlook is still concerning. A recent review noted a seven‐fold increase in 12‐year postoperative mortality amongst cirrhotic patients, emphasising the cumulative impact of liver dysfunction over time (Jacob et al., 2015). Strategies such as off‐pump CABG have shown promise in improving short‐term outcomes in these patients; however, broader, long‐term studies are needed to confirm the efficacy of these approaches (Ben Ari et al., 2006; Filsoufi et al., 2007; Hayashida et al., 2004; Kubota et al., 2013).

In light of these findings, incorporating liver function assessments into EuroSCORE II and similar risk models would offer a more accurate stratification for patients with liver disease undergoing cardiac surgery. MELD or Child–Pugh scores could be valuable additions, enabling clinicians to gauge liver‐related risks alongside cardiac risks and make more informed surgical and perioperative decisions.

### 3.2 Limitations of LVEF in assessing cardiac function

Left ventricular ejection fraction (LVEF) is a widely used measure of cardiac function and serves as a core component in the EuroSCORE II calculation for assessing surgical risk in cardiac patients. However, its limitations in accurately capturing the true extent of myocardial dysfunction are well‐documented, particularly in pathological conditions such as severe mitral regurgitation (MR) (Cikes & Solomon, 2016). While LVEF is commonly calculated using methods such as Simpson's biplane formula, which evaluates the percentage of blood ejected from the left ventricle per cardiac cycle, it is inherently a measure of chamber function rather than intrinsic myocardial contractility. Consequently, it may fail to detect subtle or early myocardial impairment that does not significantly affect global ejection.

In MR, the so‐called ‘total’ LVEF often overestimates cardiac performance due to the altered haemodynamic environment caused by the regurgitation of blood into the left atrium during systole. This regurgitant flow reduces afterload, which is defined as the resistance the left ventricle must overcome to eject blood into the aorta. The reduction in afterload lowers the myocardial wall stress during contraction, artificially preserving LVEF even when intrinsic myocardial function is compromised. Simultaneously, the increased preload – caused by the return of regurgitant blood into the left ventricle during diastole – further exaggerates LVEF through the Frank–Starling mechanism, whereby increased ventricular filling enhances stroke volume. These favourable loading conditions can mask significant myocardial damage, including the loss of contractile elements and cellular structural abnormalities, such as mitochondrial dysfunction (Suri et al., 2009, 2008). Despite the well‐recognised limitations of LVEF as an indicator of underlying myocardial function in patients with MR, risk prediction models, including EuroSCORE II have yet to incorporate more nuanced measures of myocardial function, which could enhance surgical risk stratification for this population (Dupuis et al., 2017; Enriquez‐Sarano et al., 2005; Enriquez‐Sarano & Sundt, 2010; Kang et al., 2014; Pecini et al., 2011).

Several studies have proposed alternative parameters to enhance risk stratification and surgical decision‐making in patients with MR and other cardiac conditions. Forward LVEF, for example, has demonstrated significant prognostic value in identifying patients at higher risk of adverse postoperative outcomes in this patient population (Clancy et al., 1985; Dupuis et al., 2017). Similarly, global longitudinal strain (GLS), a measure of myocardial deformation, has shown promise in detecting subclinical dysfunction even in asymptomatic patients. A meta‐analysis and systematic review found that baseline GLS independently predicts postoperative outcomes and long‐term survival in patients with severe MR and preserved LVEF (Cho et al., 2016; Clancy et al., 1985; Mascle et al., 2012). Incorporating these advanced parameters into models like EuroSCORE II could address the shortcomings of LVEF, providing a more accurate and tailored assessment of risk, optimising surgical timing and improving clinical outcomes for this complex patient population.

Heart failure with preserved ejection fraction (HFpEF) is another prevalent condition that presents significant challenges in cardiac surgery risk prediction, particularly given its under‐representation in conventional models like EuroSCORE II. Affecting up to 5.5% of the general population and approximately 24.5% of patients undergoing cardiac surgery, HFpEF is primarily characterised by diastolic dysfunction, where impaired ventricular relaxation and increased stiffness result in elevated filling pressures despite a normal ejection fraction (Nguyen et al., 2018; Oktay et al., 2013). These abnormalities, which include LV concentric hypertrophy, myocardial fibrosis and microvascular dysfunction, contribute to increased left atrial pressures and pulmonary hypertension. Unlike heart failure with reduced ejection fraction (HFrEF), these changes are not captured by LVEF or other parameters currently included in EuroSCORE II, thus potentially limiting the model's ability to assess surgical risk for this population accurately.

The limitations of LVEF in reflecting HFpEF are evident in its inability to account for diastolic dysfunction's significant impact on postoperative outcomes. A multivariate survival analysis by Nguyen et al. (2018) demonstrated that HFpEF remains an independent predictor of in‐hospital mortality in cardiac surgery patients after adjusting for EuroSCORE II. Moreover, diastolic dysfunction itself has been shown to increase mortality rates and the risk of adverse events such as renal failure, stroke, prolonged ventilation and deep sternal wound infections following surgery (Matyal et al., 2009; Merello et al., 2008). A systematic review by Kaw et al. (2016) also revealed that mortality risk escalates with the severity of preoperative diastolic dysfunction, regardless of whether LVEF is preserved (>40%) or reduced (<40%). This highlights the need for risk prediction models to move beyond reliance on LVEF as a sole marker of cardiac function.

In surgical settings, HFpEF patients are particularly vulnerable to poor outcomes due to inadequate myocardial protection during surgery, especially in those with LV hypertrophy or concentric remodelling (Duncan et al., 2008; Natsuaki et al., 2004; Orsinelli et al., 1993). These structural abnormalities increase susceptibility to ischaemic injury and perioperative complications, including difficulty in weaning from cardiopulmonary bypass (Bernard et al., 2001; Denault et al., 2006). Thus, despite preserved LVEF, patients with HFpEF face long‐term burdens comparable to those with reduced EF, including high rates of readmission, disability and persistent symptoms following hospital discharge (Smith et al., 2003). As all‐cause mortality and postoperative outcomes are influenced by diastolic dysfunction, incorporating more advanced diastolic function parameters could improve the accuracy of risk stratification. Metrics such as left atrial strain, pulmonary arterial pressures and grading of diastolic dysfunction provide valuable insights into the haemodynamic burden of HFpEF and could help refine surgical risk prediction for this high‐risk population (Boe & Smiseth, 2022; Bouwmeester et al., 2022; Hoeper et al., 2017).

### 3.3 Importance of LV dimensions in risk models

LV dimensions, a critical marker of cardiac remodelling and dysfunction, are also absent from the EuroSCORE II model despite their well‐established prognostic significance in cardiovascular disease and cardiac surgery (Aleong et al., 2015; Brown et al., 2009; Fukunaga et al., 2020; Schillaci et al., 2000; Verdecchia et al., 2001). Cardiac remodelling, characterised by changes in LV shape, dimensions, volume or mass, is a pivotal process in the progression of HFrEF. These structural changes are often accompanied by the deterioration of systolic or diastolic functions, as observed in serial imaging evaluations and are strongly associated with adverse outcomes (Yan et al., 2022). Furthermore, LV dimensions are key determinants for surgical intervention in patients with aortic regurgitation, as significant dilatation often signals progressive ventricular dysfunction and the urgent need for valve repair or replacement to prevent irreversible damage. Including LV dimensions in risk models could enhance their predictive accuracy and clinical utility (Vahanian et al., 2022).

LV hypertrophy, determined by LV mass, has long been recognised as a predictor of mortality and adverse cardiovascular events in diverse patient populations, including those with hypertension, heart failure, CAD and those undergoing CABG (East et al., 2003; Ghali et al., 1992; Schillaci et al., 2000; Verdecchia et al., 2001; Zhu et al., 2020). In patients with reduced LVEF, LV enlargement (LVE) – assessed by measuring LV end‐diastolic diameter (LVEDD) – has similarly been identified as a risk factor for adverse cardiovascular events (Narayanan et al., 2014).

Increased LV dimensions are consistently linked to poorer outcomes across various cardiac conditions and surgical contexts. Preoperative LV end‐systolic volume index exceeding 100 mL/m^2^ has been identified as a significant predictor of congestive heart failure and is associated with increased mortality in patients with ischaemic cardiomyopathy, emphasising the prognostic value of LV size in clinical decision‐making (Yamaguchi et al., 1998). These findings highlight the potential utility of incorporating LV volume or size into treatment planning.

In a more recent study, Fukunaga et al. (2020) demonstrated that patients with enlarged LV dimensions faced a 5.5‐fold higher risk of mortality and major morbidity, alongside a significantly increased likelihood of prolonged hospital stays. Similarly, Yan et al. (2022) in their multivariate analysis, identified that both LVH and LVE were independently associated with a higher risk of postoperative mortality following CABG.

Following myocardial infarction, LV dilatation often reflects irreversible structural changes, including infarct expansion, thinning of the infarcted wall and distortion of the ventricular cavity. These changes compromise ventricular efficiency and contribute to a higher incidence of postoperative complications, including prolonged ventilation, low cardiac output syndrome and difficulty in weaning from cardiopulmonary bypass (Fukunaga et al., 2020). Thus, recognising and addressing LV dimensions in risk models is critical, as their prognostic value could significantly enhance the accuracy of patient risk stratification and inform more effective surgical planning.

The EuroSCORE II model has been instrumental in cardiac surgical risk assessment, yet it fails to account for several critical cardiac‐related factors that profoundly influence surgical outcomes. Liver dysfunction, for instance, significantly increases the risk of perioperative complications and mortality, particularly in patients with advanced cirrhosis or elevated MELD scores. This parameter remains absent despite clear evidence of its impact, potentially underestimating risk for a considerable subset of patients. Similarly, the reliance on LVEF as a primary measure of cardiac function overlooks its limitations in MR and HFpEF. Furthermore, LV dimensions, long recognised as markers of cardiac remodelling and adverse outcomes, remain unaddressed in the current model, diminishing their ability to identify high‐risk patients.

Addressing these gaps by incorporating liver function assessments, refined myocardial performance metrics and LV dimensions into risk prediction models would provide a more comprehensive and accurate evaluation of surgical risk. Such enhancements would better capture the intricate interplay of cardiac and systemic factors, enabling clinicians to optimise perioperative strategies and improve patient outcomes. Aligning the model with contemporary cardiac surgical practice realities is essential to ensure it remains a relevant and reliable tool for guiding clinical decision‐making.

## 4 OPERATION‐RELATED FACTORS

### 4.1 Differentiating procedure‐specific risks

While the current EuroSCORE II framework has been designed to classify interventions based on their extent and complexity, the current categorisation oversimplifies the technical challenges and risks associated with various procedures. This lack of granularity undermines the accuracy of risk stratification and diminishes the system's utility in guiding clinical decision‐making and benchmarking surgical outcomes.

One significant limitation is the lack of differentiation between procedures with distinct risk profiles. For instance, mitral valve repair is well‐recognised as less risky than replacement, primarily due to preserving the subvalvular apparatus, which maintains ventricular geometry and function. Despite this, repair and replacement are grouped under the current scoring system. This approach does not acknowledge the substantial differences in technical demands and long‐term outcomes. In contrast, the STS scoring system explicitly distinguishes between mitral valve repair and replacement by assigning distinct risk weights to each procedure, providing a more accurate reflection of their risks and better supporting surgical planning (Fu et al., 2021; Gillinov et al., 2008; Saurav et al., 2015; Zhou et al., 2010).

The oversimplification extends to the classification of combined procedures. In the current framework, combinations of CABG with valvular procedures are treated as a single group despite the variability in risk depending on the specific procedures involved. This contrasts significantly with the STS system, which provides more refined assessments. For instance, it distinguishes between CABG performed alongside mitral valve repair and CABG with ascending aortic replacement. This level of specificity may allow for a more comprehensive understanding of the additive risks involved in these combinations.

Another significant deficiency is the omission of specific high‐risk procedures that should be highlighted due to their inherently greater technical difficulty and higher risk of adverse outcomes. Pericardiectomy, for example, is a challenging operation often performed on patients with constrictive pericarditis, a condition associated with severe haemodynamic compromise and multiple comorbidities. This procedure carries a considerably higher risk than many isolated valve surgeries, particularly amongst those with radiation pericarditis and associated myocardial disease, but it is not distinctly categorised within the current framework (Busch et al., 2015; Piehler et al., 1985; Syed et al., 2014). By failing to account for such procedures, the EuroSCORE II system potentially underestimates the risk in cases requiring exceptional technical expertise.

### 4.2 Accounting for CAD complexity

In addition, the EuroSCORE II model also inadequately accounts for the complexity and severity of CAD, a key determinant of outcomes in cardiac surgery. The model's binary treatment of CAD, without distinguishing between single‐vessel disease (SVD), multi‐vessel disease (MVD) or left main stem (LMS) stenosis, overlooks critical anatomical and pathological differences that significantly influence surgical risk and long‐term prognosis.

It is well‐established that patients with extensive CAD, such as LMS stenosis combined with MVD, face disproportionately higher risks compared to those with SVD or LMS alone. For instance, the SYNTAX trial demonstrated a higher rate of major adverse cardiac or cerebrovascular events in patients with LMS disease and two‐ or three‐vessel involvement than in those with LMS combined with SVD (Serruys et al., 2009). Furthermore, higher SYNTAX scores, which quantify lesion complexity, are associated with poorer outcomes in both CABG and percutaneous coronary intervention Patients with three‐vessel disease, for example, exhibit a threefold increased risk of mortality compared to those with SVD (Lopes et al., 2008). These differences in risk are amplified in cases involving severe calcification, bifurcation lesions or diffuse disease, which pose significant technical challenges during revascularisation and increase the likelihood of complications such as incomplete revascularisation, graft failure or residual ischaemia.

The omission of CAD complexity from EuroSCORE II may lead to an underestimation of surgical risk, particularly in high‐risk populations. While the presence of CAD is considered in the model, qualitative and quantitative differences – such as the number of diseased vessels, lesion location and anatomical challenges – are overlooked. It was shown in the study by Beach et al. that LMS stenosis ≥70%, proximal Left anterior descending artery stenosis ≥70% or significant right coronary artery or left circumflex stenosis have all been associated with worse outcomes in patients undergoing aortic valve replacement and CABG. Nevertheless, these factors remain absent from the risk calculation (Beach et al., 2013). Likewise, in a study of 6151 patients undergoing aortic valve replacement plus CABG, those with LMS or three‐vessel CAD had significantly higher rates of postoperative complications – such as prolonged ventilation, stroke and renal failure – compared to those with one‐ or two‐vessel CAD, even though this did not result in higher operative or 1‐year mortality (Li et al., 2013).

The current EuroSCORE II model falls short of accurately reflecting the complexity of modern cardiac surgery by oversimplifying key risk factors. It fails to differentiate between mitral valve repair and replacement procedures or adequately account for the varying risks of combined operations, such as CABG with valvular interventions. Additionally, the model overlooks the severity and extent of CAD, as well as the challenges posed by high‐risk procedures like pericardiectomy. By incorporating these factors – along with procedural distinctions, CAD complexity and lesion characteristics – future iterations of EuroSCORE II could achieve greater predictive accuracy and clinical relevance. These enhancements would ensure more precise risk stratification, aligning the model with the realities of contemporary surgical practice, ultimately improving patient outcomes and supporting better‐informed clinical decision‐making.

## 5 REVALIDATION OF EuroSCORE II

EuroSCORE II has long been a fundamental tool in cardiac surgical risk stratification, supporting clinical decision‐making and outcome prediction. However, as cardiac surgery becomes more complex due to changes in patient demographics, advancements in surgical techniques, evolving disease identification, and improvements in perioperative care and patient management, there is a clear need to refine and adapt the model to maintain its relevance and accuracy. Its predictive limitations stem not only from the omission of certain key factors but also from the oversimplification of others, including patient‐related (frailty, ethnicity, IE), cardiac‐related (liver dysfunction, LV function, LV dimension) and operation‐related variables (weight of operation, CAD complexity). Refining these variables or incorporating additional ones could improve predictive accuracy.

Machine learning (ML) has emerged as a promising avenue for enhancing or complementing traditional risk models. Unlike conventional logistic regression, which underpins EuroSCORE II, ML techniques can process large datasets and account for complex, non‐linear relationships between variables (Nashef and Ali, 2023). ML‐based models can offer the potential for more precise surgical risk assessment by accommodating more granular patient data and refining predictions with continuous learning.

Recent studies suggest that ML may outperform EuroSCORE II in predicting postoperative mortality. For instance, Allyn et al. (2017) compared ML models with EuroSCORE II in elective cardiac surgery and found that ensemble ML techniques, which integrate multiple ML algorithms, provided greater predictive accuracy. Additionally, ML models can identify interactions between variables that conventional approaches may overlook, such as the combined impact of frailty, ethnicity and comorbidities on surgical outcomes (Elfanagely et al., 2021; Sajeev et al., 2022).

Despite these advantages, ML adoption in clinical practice requires rigorous validation across diverse populations to ensure reliability and equity. A hybrid approach integrating ML with existing EuroSCORE II parameters may best balance advanced analytics and clinical usability. This method would enhance the model's ability to predict outcomes while maintaining the familiarity and interpretability clinicians rely on in current practice.

## 6 CONCLUSION

EuroSCORE II must evolve to stay clinically relevant by refining existing variables and incorporating additional patient‐, cardiac‐ and operation‐related factors to enhance its predictive accuracy. Leveraging ML could be the next step forward, enabling a more adaptive and precise ‘EuroSCORE III’ that enhances risk stratification, optimises perioperative management and improves patient outcomes in modern cardiac surgery.

## AUTHOR CONTRIBUTIONS

Wael I. Awad conceptualized and correcting manuscript. Jing Yong Ng first draft. Remining authors checked and improved manuscript.

## CONFLICT OF INTEREST

D.M.B. is Editor‐in‐Chief of *Experimental Physiology*, Chair of the Life Sciences Working Group, member of the Human Spaceflight and Exploration Science Advisory Committee to the European Space Agency and member of the Space Exploration Advisory Committee to the UK and Swedish National Space Agencies. D.M.B. is also affiliated to Bexorg, Inc. (USA), focused on the technological development of novel biomarkers of cerebral bioenergetic function and structural damage in humans.

## Data Availability

The evidence used to support this review is publicly available in electronic databases such as PubMed, Ovid, Scopus and Embase.
